# Obesity Is Associated with a Lower Risk of Mortality and Readmission in Heart Failure Patients with Diabetes

**DOI:** 10.3390/biomedicines13123086

**Published:** 2025-12-15

**Authors:** Rayane El-Khoury, Ziyad Mahfoud, Soha Dargham, Mujtaba Ashal Pal, Amin Jayyousi, Jassim Al Suwaidi, Charbel Abi Khalil

**Affiliations:** 1 Department of Genetic Medicine, Weill Cornell Medicine-Qatar, Doha P.O. Box 24144, Qatar; 2 Biostatistics Core, Weill Cornell Medicine-Qatar, Doha P.O. Box 24144, Qatar; 3 Department of Medical Education, Weill Cornell Medicine-Qatar, Doha P.O. Box 24144, Qatar; 4 Department of Endocrinology, Hamad Medical Corporation, Doha P.O. Box 3050, Qatar; 5 Heart Hospital, Hamad Medical Corporation, Doha P.O. Box 3050, Qatar; 6 Joan and Sanford I. Weill Department of Medicine, Weill Cornell Medicine, New York, NY 10065, USA

**Keywords:** obesity, heart failure, BMI, type 2 diabetes, cardiovascular disease, readmission, mortality, NRD database

## Abstract

**Objectives:** We aim to investigate the relationship between body weight and the risk of cardiovascular events in heart failure patients with diabetes. **Methods:** We therefore conducted a retrospective analysis of HF patients with T2D using the Nationwide Readmissions Database (NRD) from 2016 to 2022. Patients were stratified by BMI classes: underweight, normal weight, overweight, and obesity classes I–III. The primary outcome was in-hospital mortality. Secondary outcomes included 1-year mortality and readmission for heart failure. **Results:** A total of 26,199 patients with BMI data were included in the analysis. Underweight patients had the highest risk of in-hospital mortality [aOR = 1.80 (95% CI: 1.16–2.80)] and cardiogenic shock [aOR = 2.13 (95% CI: 1.26–3.59)]. In contrast, obesity classes I–III were associated with significantly lower odds of those events. One-year mortality rates did not differ significantly across BMI groups. However, obesity classes II and III were associated with a lower adjusted risk of HF readmission [aHR = 0.71 (95% CI: 0.50–0.99); aHR = 0.68 (95% CI: 0.49–0.96), respectively]. **Conclusions:** In patients with T2D and HF, an obesity paradox exists whereby patients with obesity have a lower risk of in-hospital mortality and cardiogenic shock. Further, obesity classes II-III are associated with a lower risk of 1-year readmission for HF.

## 1. Introduction

The prevalence of heart failure (HF) is rising worldwide. In 2023, it was estimated that approximately 64 million people were affected by HF globally [1,2]. Patients with T2D are approximately twice as likely to develop HF as those without diabetes [3]. Further, the prevalence of T2D in patients with HF has been reported to be as high as 39% [4]. Furthermore, T2D is associated with worse outcomes in patients with established HF [5].

The “obesity paradox” describes the counterintuitive finding that, despite being a major cardiovascular risk factor, obesity may be associated with improved survival once cardiovascular disease is present [6]. An obesity paradox has been reported in several cardiovascular diseases, such as diabetes [7], myocardial infarction [8], chronic kidney disease (CKD) [9], and hypertension [10].

Obesity is well-established as an independent risk factor for developing HF, specifically with preserved ejection fraction (HFpEF) [11,12]. It is estimated that more than 80% of patients with HFpEF but less than 50% of those with reduced ejection fraction (HFrEF) have concomitant obesity [13,14,15,16]. Despite this, an obesity paradox has been described in HF, where overweight or obese patients exhibit lower mortality rates compared with those of normal weight [17]. This study aims to investigate the relationship between body weight and the risk of cardiovascular events in heart failure patients with diabetes.

## 2. Methods

### 2.1. Data Source

The study used patient data from the Nationwide Readmissions Database (NRD) for the years 2016 to 2022. The NRD collects hospital data from the United States and contains de-identified patient information, including clinical characteristics, hospital discharges, and readmissions. Diagnoses are encoded according to their respective International Classification of Diseases, 10th Revision (ICD-10) coding system. Access to the NRD database was granted through a data use agreement between the Healthcare Cost and Utilization Project (HCUP) and Weill Cornell Medicine-Qatar. The study received administrative IRB approval (record number 21-0001) and complied with both local and international ethical standards. Patient consent was not required due to the retrospective and de-identified nature of the data. This study was conducted and reported in accordance with the Strengthening the Reporting of Observational Studies in Epidemiology (STROBE) guidelines (Table A1).

### 2.2. Patients and Outcomes

Patients were included if they had a primary diagnosis of heart failure (ICD-10 codes: I5020, I5021, I5022, I5023, I5030, I5031, I5032, and I5033) and a secondary diagnosis of type 2 diabetes (ICD-10: E08x, E09x, E10x, E11x, E13x, O24.1x, O24.3x, O24.8, O24.9). Individuals under 18 years of age were excluded, as were cases with missing data. Patients were stratified by BMI classes. These BMI classes were defined as: Underweight (BMI < 19.9 kg/m^2^), Normal weight (BMI: 20.0–24.9 kg/m^2^), Overweight (BMI: 25.0–29.9 kg/m^2^), obesity class I (BMI: 30.0–34.9 kg/m^2^), class II (BMI:35.0–39.9 kg/m^2^), and class III (BMI ≥ 40 kg/m^2^). The primary outcome was in-hospital mortality. Secondary outcomes included cardiogenic shock, ventricular fibrillation (VFIB), atrial fibrillation (AFIB), and acute renal failure (ARF), and 1-year mortality and readmission for heart failure. All ICD-10 coding used is provided in Table A2.

### 2.3. Analysis Plan and Statistics

Data collected from all study years were merged for analysis. Baseline patient characteristics and relevant outcomes were evaluated and compared across all BMI classes. Descriptive statistics were reported as frequencies and percentages for categorical variables, and as mean (SD) or median (IQR) for continuous variables, where appropriate. Logistic regression models were used to estimate odds ratios (ORs) and their 95% confidence intervals (CIs). Adjusted analyses were reported as adjusted odds ratios (aORs) with their corresponding 95% CIs. Multivariable logistic models were adjusted for BMI, age, sex, income level, coronary artery disease (CAD), hypertension, smoking status, dyslipidemia, peripheral vascular disease (PVD), and CKD. Kaplan–Meier (KM) curves were generated to visualize time-to-event data and to compare the groups’ cumulative 1-year mortality and heart failure risk. A log-rank test was used to compare outcomes, and a Cox proportional hazard model was used to adjust for different baseline characteristics. The results were presented as hazard ratios (HR), adjusted hazard ratios (aHR), and their respective 95% CIs. Significant HRs with a *p*-value cutoff of 0.2 were eligible for inclusion into the adjusted HR model. One-year mortality was defined as death occurring within one year of a previous hospital discharge, among patients who were readmitted alive. It represented the time from the prior hospitalization to the subsequent death. The goodness-of-fit of the models was assessed using the Hosmer–Lemeshow test. Calibration graphs and regressions were used to assess how well the model predicts cardiovascular events. A *p*-value ≤0.05 was considered statistically significant. All analyses were performed using Stata/SE version 17.0 (StataCorp, College Station, TX, USA).

## 3. Results

### 3.1. Study Population

**Figure 1:**
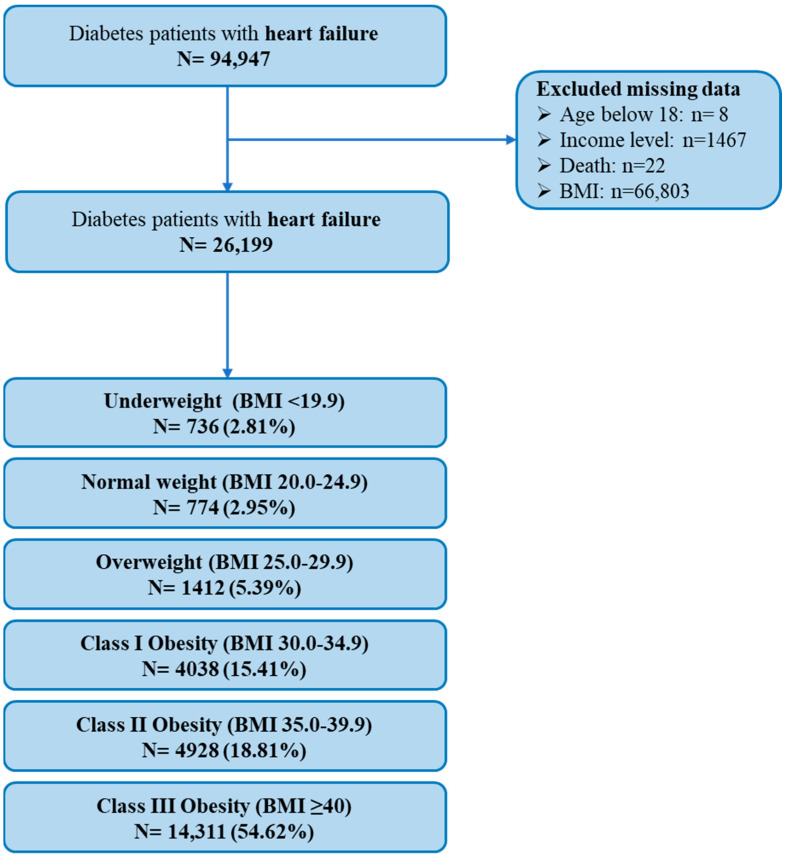
Flowchart of the study.

A total of 94,499 heart failure patients with type 2 diabetes were identified. After excluding patients with missing BMI or mortality data, 26,199 patients were included in the final analysis (Figure 1).

### 3.2. Baseline Characteristics

Baseline characteristics of heart failure patients with diabetes are available in Table A3. Those with missing BMI data were older, more likely to be males, and had a higher prevalence of CAD, hypertension, and CKD (*p* < 0.05 for all). In patients with available BMI data, the mean age decreased with increasing BMI classification, from 78 years among underweight patients to 63 years among those with obesity class III (*p* < 0.001) (Table 1). Across all BMI categories, the majority of patients originated from low-income households. Interestingly, the prevalence of CAD, PVD, and CKD was the lowest among patients with obesity class III compared to other BMI classes. Hypertension was consistently high in all patients, affecting over 60% of them and up to 70% in obesity class I patients. Dyslipidemia and smoking were more prevalent in patients with obesity class I.

### 3.3. In-Hospital Outcomes

Table 2 presents the adjusted and unadjusted logistic regression of in-hospital outcomes across different BMI categories. A U-shaped curve governs the relation between BMI and in-hospital mortality, illustrating the obesity paradox. Underweight patients exhibited higher adjusted odds of mortality [aOR = 1.81 (95% CI: 1.16–2.81)].

**Figure 2:**
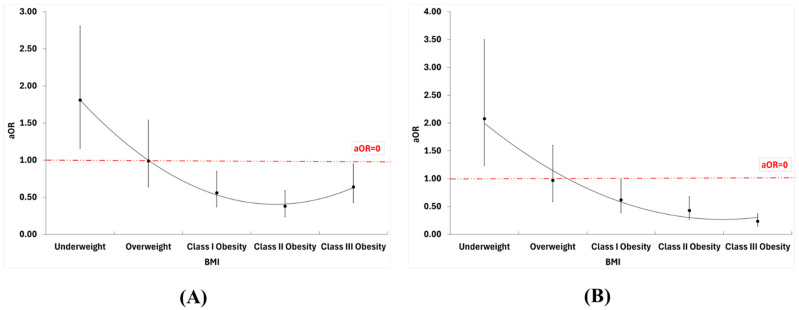
Adjusted odds ratio for (**A**) mortality and (**B**) cardiogenic shock across different BMI classes.

Patients with obesity classes I, II, and III have a lower adjusted odds ratio (aOR) compared to patients with a normal BMI range (Figure 2A), suggesting potential protective effects of higher BMI in patients with heart failure and type 2 diabetes. A more pronounced downward trend governs the relationship between BMI and cardiogenic shock, without the upward inflection in obesity class III seen in mortality (Figure 2B). Underweight patients had increased adjusted odds of cardiogenic shock [aOR = 2.08 (95% CI: 1.24–3.50)], whereas the risk decreased with increasing BMI categories. Furthermore, the underweight population was associated with lower odds of AFIB [aOR = 0.78 (95% CI: 0.63–0.96)] while obesity class III was found to have a significantly increased risk [aOR = 1.39 (95% CI: 1.19–1.62)] compared to normal weight. We did not observe an impact of BMI on ventricular fibrillation or acute renal failure.

Several predictors were independently associated with mortality and cardiogenic shock. As expected, age increased the risk of mortality [aOR = 1.04 (95% CI 1.03–1.05)]. The female gender was associated with a 20% lower risk of mortality and a 36% lower risk of cardiogenic shock. CAD and CKD increased the risk of both outcomes. In contrast, hypertension conferred protection against mortality [aOR = 0.72 (95% CI: 0.58–0.90)] and cardiogenic shock [aOR = 0.50 (95% CI: 0.40–0.62)] (Table A4).

### 3.4. 1-Year Outcomes

**Figure 3:**
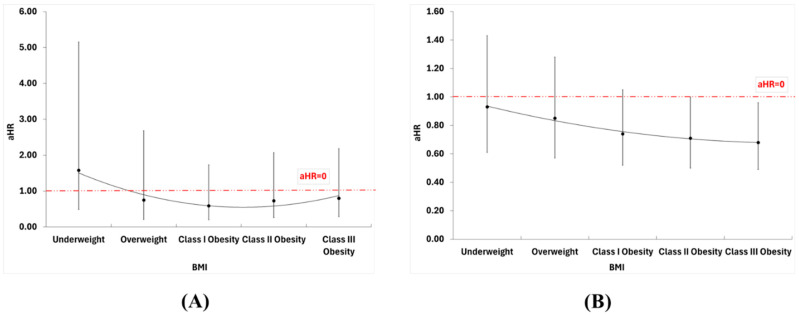
Cox Proportional Hazards models for (**A**) 1-year mortality and (**B**) readmission for HF.

Among the 26,999 initially hospitalized patients, 487 (1.8%) died, and 25,712 (95.2%) were discharged. Of the 11,278 readmitted patients, 6829 had BMI data available and were subsequently followed up for one calendar year. Compared to normal weight, obesity class I [HR = 0.41 (95% CI: 0.14–1.19)], obesity class II [HR = 0.48 (95% CI: 0.17–1.36)], and obesity class III [HR = 0.46 (95% CI: 0.17–1.25)] were not associated with a statistically significant lower risk of 1-year mortality. The results were unchanged after adjustment (Table A5, Figure 3A).

Compared to the normal BMI range, class II and class III obesity were significantly associated with a lower risk of readmission for heart failure [HR = 0.70 (95% CI: 0.50–0.99); and HR = 0.65 (95% CI: 0.47–0.91), respectively]. In the adjusted model, BMI associations remained similar, with class II obesity linked to a 29% reduced hazard [aHR = 0.71 (95% CI: 0.50–1.00)] and class III obesity to a 32% reduced hazard [aHR = 0.68 (95% CI: 0.49–0.96)] (Table A5, Figure 3B). Significant unadjusted predictors included age [HR = 1.01 (95% CI: 1.00–1.02)], hypertension [HR = 1.36 (95% CI: 1.22–1.51)], and PVD [HR = 1.11 (95% CI: 1.02–1.21)].

### 3.5. Goodness of Fit and Calibration

**Figure A1:**
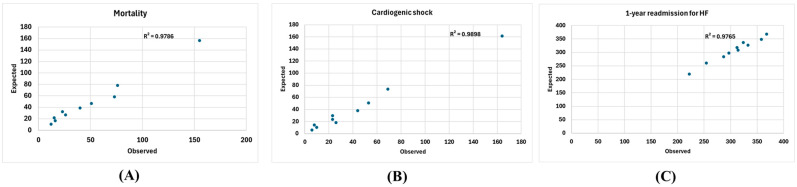
Goodness of fit for (**A**) mortality, (**B**) cardiogenic shock, and (**C**) 1-year readmission for heart failure.

The *p*-values of the Hosmer–Lemeshow goodness-of-fit test were all not significant for in-hospital mortality, cardiogenic shock, and 1-year heart failure readmission (*p*-values 0.3161; 0.3579; and 0.9615, respectively). This indicates that the data fit the model. In addition, the R^2^ values for the calibration curves are 0.9786, 0.9898, and 0.9765 (Figure A1). These high R^2^ values indicate that the models are excellent at predicting cardiovascular outcomes.

## 4. Discussion

In this large US administrative database, we showed that obesity is associated with a lower risk of in-hospital mortality in heart failure patients with diabetes. Further, obesity is associated with a reduced risk of heart failure within 1 year of discharge. In contrast, underweight patients exhibited the highest risk of in-hospital mortality and cardiogenic shock, supporting the presence of an “obesity paradox”.

### 4.1. Obesity Paradox

To the best of our knowledge, we are the first to report an obesity paradox in heart failure patients with diabetes. However, our findings are consistent with previous studies demonstrating an obesity paradox in the general heart failure population or in patients with diabetes. A systematic review of nine heart failure cohorts reported a better cardiovascular outcome in overweight and obese patients, except in those with a BMI > 40 kg/m^2^ [18]. A recent analysis of HF patients followed at a primary healthcare setting in the UK also reported a U-shaped relationship between BMI and long-term all-cause mortality [19]. In contrast, obesity did not have an impact on 1-year mortality and readmission in HF patients treated with angiotensin-receptor neprilysin inhibitors (ARNIs) [20]. Further, we demonstrated that obesity is associated with a lower risk of cardiogenic shock, which aligns with recent data from a Korean cardiogenic shock registry reporting lower mortality in these patients [21]. Furthermore, we observed that individuals with severe obesity had a higher risk of developing AFIB, reflecting the global rise in AFIB closely linked to the obesity epidemic [22]. In a 15-year follow-up of an Australian cohort, each 4.2 kg/m^2^ increase in BMI was associated with a 16% higher risk of AFIB in women and a 64% higher risk in men [23].

An obesity paradox has also been reported in patients with type 2 diabetes. In a large prospective cohort that included around 10,000 patients from the National Health Service, Costanzo et al. reported a lower risk of mortality in overweight patients with type 2 diabetes [24]. Interestingly, the risk of mortality was not significantly higher in obese patients despite the higher incidence of cardiovascular events. The analysis of the Swedish Renal Registry revealed that underweight patients with diabetic nephropathy had a higher risk of long-term mortality. In contrast, individuals with obesity were less likely to die, except those on dialysis [25]. We have recently demonstrated that overweight and obese diabetes patients hospitalized for ST-elevation myocardial infarction have a higher incidence of in-hospital mortality [8]. Interestingly, Park et al. showed that in Korean patients with acute coronary syndrome, the U-shape relation between BMI and cardiovascular outcomes was less significant in patients with type 2 diabetes [26].

While it is already known that hypotension is associated with poor outcomes in acute and chronic HF patients [27], our analysis showed that hypertension is associated with approximately 25% lower risk of mortality. A systematic review of 10 studies on HF and blood pressure (BP) reported a 13% lower risk for every 10 mmHg increase in systolic BP above 125 mmHg [28]. Impaired myocardial contractility may limit the ability to sustain normal arterial pressure in HF patients; thus, low BP often reflects advanced disease severity and reduced cardiac reserve [29]. Conversely, high BP may indicate a less severe stage of the disease with better hemodynamic compensation. We also showed that females had a lower mortality risk, which suggests that gender may act as an effect modifier. It is already known that women are more likely to have HFpEF than HFrEF [30], the latter being associated with higher mortality risk [31]. Other plausible mechanisms include the cardioprotective influence of estrogen, with residual hormonal effects persisting even after menopause [32]. Women also tend to accumulate more subcutaneous rather than visceral adipose tissue [33], the latter being metabolically more detrimental. In addition, circulating levels of adiponectin—a protective adipokine—are generally higher in women and may contribute to improved outcomes [34].

### 4.2. Pathophysiology

The obesity paradox may be explained by several mechanisms: Obese patients have greater metabolic and nutritional reserves that mitigate catabolic stress [19,35,36,37]. In contrast, underweight status often reflects cardiac cachexia, frailty, and comorbidities such as chronic kidney disease and malnutrition, all of which contribute to poorer outcomes [38]. It may also be that the expansion of adipose tissue in metabolically healthy obese patients has direct cardioprotective effects, such as improved coronary perfusion and reduced oxygen demand [39]. One of the plausible explanations of the obesity paradox is the immune function modulation in obese patients [40]. HF patients experience impaired immune defense and heightened vulnerability to stressors, infections, and HF progression [41]. Additional factors include lower sympathetic activation and norepinephrine levels [42]. Farre et al. showed that obese chronic HF patients have lower baseline levels of norepinephrine compared to patients with a normal BMI; hence, a decreased sympathetic activation could contribute to the obesity paradox [42]. The protective effect of obesity also appears to be limited in severe obesity, likely due to a greater comorbidity burden and restricted physical activity. Furthermore, evidence suggests a J-shaped association between BMI and arrhythmias, where obesity increases the risk of atrial fibrillation and ventricular tachyarrhythmias [43,44]. At the same time, underweight is linked to higher rates of cardiogenic shock [21,23,45]. These potential mechanisms remain speculative, as they were not directly evaluated in the present study, and therefore should be interpreted as hypothesis-generating.

### 4.3. Limitations

#### 4.3.1. Selection Bias and Missing Parameters

Several limitations should be noted. The NRD is a retrospective administrative database, which limits the ability to make causal inferences. Further, the database does not permit follow-up beyond a calendar year, thereby limiting the evaluation of long-term and cause-specific outcomes. The definition of cardiovascular outcomes relied solely on ICD-10 codes in the absence of clinical/lab criteria, which could affect internal validity. The NRD database also lacks several key parameters relevant to our analysis, such as medications, left ventricular ejection fraction, diabetes control, post-hospitalization diagnoses, and disease duration. The recent decade has witnessed significant progress in the treatment of heart failure. For instance, Angiotensin Receptor-Neprilysin Inhibitors (ARNIs) significantly improved mortality in patients with HFrEF and reduced the risk of heart failure hospitalization [46]. Recent diabetes therapies such as SGLT-2 inhibitors and GLP-1 receptor agonists are well established to provide cardioprotective benefits, particularly in HF patients [47]. However, we were unable to evaluate whether the use of these agents differed across BMI categories in our cohort. Accounting for these treatments, along with other missing clinical parameters, could have influenced the study outcomes, particularly with respect to heart failure phenotyping. Approximately 70% of the initial dataset lacked BMI classification, leading to the exclusion of many patients and creating a selection bias. BMI ranges were derived from ICD-10 coding; therefore, we could not rule out misclassification. Further, BMI does not capture body fat distribution or muscle mass, which may substantially influence the interpretation of the obesity paradox [48]. Furthermore, we couldn’t rule out that the excess weight in patients with overweight and obesity is secondary to fluid congestion. Most importantly, it is not known whether weight and, subsequently, BMI were measured at admission, discharge, or at random, which could influence the outcome, knowing that body-weight fluctuations are independently associated with increased risks of cardiovascular events in HF patients [49]. Finally, the marked imbalance in sample size across BMI categories, particularly the very small underweight group (<2% of the total cohort), may have reduced statistical power. This could contribute to less precise estimates and lower statistical significance in some comparisons, especially within the underweight subgroup.

In this study, 1-year mortality was defined as death occurring during hospital readmissions; therefore, out-of-hospital deaths were not captured, as linkage to death registries was not available in the NRD. The analysis of 1-year outcomes was limited to patients who were readmitted and had available BMI data, which represents only a subset of the full database. This restriction may reduce the generalizability of the results, as it excludes patients who were never readmitted or who died outside of a hospital. As a result, the reported mortality and readmission rates may underestimate the true incidence, and the observed associations between BMI and long-term outcomes should be interpreted cautiously. Future studies with more complete follow-up, including out-of-hospital deaths, are needed to validate these findings.

#### 4.3.2. COVID-19 Pandemic

The study period (2016–2022) overlaps with the COVID-19 pandemic from 2020 to 2022, which was not accounted for in our study. During the pandemic, hospital admissions, in-person patient care, and follow-up strategies for patients with HF changed substantially [50,51,52]. Studies have reported a decline in HF hospitalizations early on in the pandemic, with admitted patients having more severe outcomes [53,54,55,56], which could have impacted HF-related admissions, length of stay, and readmission rates [57]. In parallel, there was a rapid expansion of telemedicine and remote monitoring, which introduced new approaches to HF management [58] that are also not captured by the NRD. Non-face-to-face follow-up for HF care may have influenced post-discharge outcomes [59].

### 4.4. Strengths and Generalizability

A major strength of this study is the use of a large, nationally representative inpatient database that includes millions of patients and over 25,000 HF patients with BMI data, which allows robust multivariable adjustment and comprehensive sensitivity analyses. This enables the generalizability of our findings to the US population. The NRD database captures readmissions within the calendar year, making it well-suited for evaluating 1-year outcomes. The use of standardized ICD-10 codes for BMI and comorbidities allowed for consistent patient stratification and improved comparability across subgroups. Furthermore, the application of both logistic and Cox regression models, along with high model performance (R^2^ > 0.97), supports the internal validity and reliability of our results.

Finally, weight loss is associated with remission of type 2 diabetes in the general population [60] and a lower incidence of cardiovascular events in the long term, as reported in a post hoc analysis of the Look AHEAD trial [61]. Although unintentional weight loss is associated with higher mortality in heart failure patients [62], it is not known whether intentional weight loss is beneficial for obese heart failure patients with diabetes. While GLP-1 agonists are indicated in obese patients with type 2 diabetes, their role is less clear in those with heart failure. Initial data suggest a cardiovascular protective effect of these medications in patients with HFpEF [63]. However, an alarming increase in the risk of HF hospitalizations has been recently demonstrated [64].

## 5. Conclusions

In HF patients with T2D, an obesity paradox exists whereby patients with obesity have a lower risk of in-hospital mortality and cardiogenic shock. Still, those who are underweight have a higher risk. Further, class II-III obesity is associated with a lower risk of 1-year readmission for HF, but not mortality. Our findings suggest an association between body weight and outcomes in patients with heart failure and diabetes. While insightful, the findings are preliminary and warrant further validation in adequately powered, prospective investigations.

## Figures and Tables

**Table 1:** Baseline characteristics of heart failure patients with diabetes across different BMI classes.

	Underweight *N* (%)	Normal Weight *N* (%)	Overweight *N* (%)	Class I Obesity *N* (%)	Class II Obesity *N* (%)	Class III Obesity *N* (%)	*p*-Value ^1^
	736 (2.81%)	774 (2.95%)	1412 (5.39%)	4038 (15.41%)	4928 (18.81%)	14,311 (54.62%)	
Age							
Mean (SD)	77.92 (11.21)	76.01 (12.13)	73.42 (11.90)	69.95 (12.30)	67.27 (12.37)	63.65 (12.77)	<0.001
Gender							
Male	330 (44.84)	437 (56.46)	776 (54.96)	2184 (54.09)	2637 (53.51)	6099 (42.62)	<0.001
Income ^2^							<0.001
Low	239 (32.47)	245 (31.65)	445 (31.52)	1356 (33.58)	1642 (33.32)	5201 (36.34)	<0.001
Low-middle	181 (24.59)	194 (25.06)	395 (27.97)	1123 (27.81)	1362 (27.64)	4156 (29.04)	
Middle-High	179 (24.32)	179 (23.13)	327 (23.16)	927 (22.96)	1157 (23.48)	3150 (22.01)	
High	137 (18.61)	156 (20.16)	245 (17.35)	632 (15.65)	767 (15.56)	1804 (12.61)	
Comorbidities							
CAD	378 (51.36)	425 (54.91)	824 (58.36)	2172 (53.79)	2391 (48.52)	5162 (36.07)	<0.001
Hypertension	470 (63.86)	511 (66.02)	1093 (77.41)	3100 (76.77)	3839 (77.90)	10,678 (74.61)	<0.001
Smoking	293 (39.81)	303 (39.15)	608 (43.06)	1896 (46.95)	2219 (45.03)	5638 (39.40)	<0.001
Dyslipidemia	308 (41.85)	374 (48.32)	777 (55.03)	2239 (55.45)	2607 (52.90)	6563 (45.86)	<0.001
PVD	267 (36.28)	253 (32.69)	463 (32.79)	1081 (26.77)	1191 (24.17)	2422 (16.92)	<0.001
CKD	199 (27.04)	256 (33.07)	491 (34.77)	1253 (31.03)	1500 (30.44)	3618 (25.28)	<0.001
Hospital Course							
Length of stay (IQR days)	5 (3–8)	5 (3–9)	4 (3–7)	4 (3–6)	4 (3–6)	4 (3–7)	<0.001

^1^ *p*-values for continuous variables were calculated using one-way ANOVA and for categorical variables using the chi-square test. ^2^ Income level was pre-coded in the NRD as “ZIPINC_QRTL”, representing median household income for the patient’s ZIP Code, categorized into four quartiles (Q1 = lowest, Q4 = highest). Cutoffs are updated annually. Additional details are available at: https://hcup-us.ahrq.gov/db/vars/zipinc_qrtl/nrdnote.jsp (accessed on 1 June 2025). BMI classes are defined according to the ICD-10 classification as: Underweight (BMI < 19.9 kg/m^2^), Normal (BMI: 20.0–24.9 kg/m^2^), Overweight (BMI: 25.0–29.9 kg/m^2^), Class I Obesity (BMI: 30.0–34.9 kg/m^2^), Class II Obesity = (BMI: 35.0–39.9 kg/m^2^), and Class III Obesity (BMI ≥ 40 kg/m^2^). Abbreviations: CAD = coronary artery disease, CKD = chronic kidney disease, PVD = peripheral vascular disease.

**Table 2:** Number of events and unadjusted odds ratios of outcomes across different BMI classes among heart failure patients with diabetes.

	Mortality			Cardiogenic Shock			Ventricular Fibrillation			Atrial Fibrillation			Acute Renal Failure		
	*n*	OR	aOR	*n*	OR	aOR	*n*	OR	aOR	*n*	OR	aOR	*n*	OR	aOR
		(95% CI)	(95% CI)		(95% CI)	(95% CI)		(95% CI)	(95% CI)		(95% CI)	(95% CI)		(95% CI)	(95% CI)
Underweight	57	1.83	**1.81**	40	1.72	**2.08**	6	2.11	2.38	322	0.83	**0.78**	167	0.81	0.90
		(1.18–2.83)	**(1.16–2.81)**		(1.03–2.87)	**(1.24–3.50)**		(0.53–8.48)	(0.59–9.61)		(0.68–1.02)	**(0.63–0.96)**		(0.64–1.03)	(0.70–1.15)
Normal weight ^†^	34	Ref	Ref	25	Ref	Ref	3	Ref	Ref	374	Ref	Ref	205	Ref	Ref
Overweight	53	0.84	0.99	47	1.03	0.97	10	1.83	1.60	597	0.78	0.90	376	1.01	0.99
		(0.55–1.32)	(0.64–1.54)		(0.63–1.69)	(0.59–1.60)		(0.50–6.68)	(0.44–5.88)		(0.66–0.93)	(0.75–1.08)		(0.83–1.23)	(0.80–1.23)
Class I Obesity	74	0.41	**0.56**	90	0.68	**0.62**	7	0.45	0.39	1689	0.77	1.08	971	0.88	0.92
		(0.27–0.61)	**(0.37–0.85)**		(0.44–1.07)	**(0.39–0.99)**		(0.12–1.73)	(0.10–1.51)		(0.66–0.90)	(0.92–1.27)		(0.74–1.05)	(0.76–1.11)
Class II Obesity	54	0.24	**0.38**	83	0.51	**0.43**	9	0.47	0.37	1977	0.82	1.16	1153	0.85	0.90
		(0.16–3.73)	**(0.24–0.59)**		(0.33–0.81)	**(0.27–0.68)**		(0.13–1.74)	(0.10–1.41)		(0.62–0.83)	(0.98–1.36)		(0.71–1.01)	(0.75–1.08)
Class III Obesity	215	0.33	**0.64**	141	0.30	**0.24**	29	0.52	0.41	5361	0.64	**1.39**	3213	0.80	0.96
		(0.23–0.48)	**(0.43–0.95)**		(0.19–0.46)	**(0.15–0.37)**		(0.16–1.72)	(0.12–1.40)		(0.55–0.74)	**(1.19–1.62)**		(0.68–0.95)	(0.80–1.15)

BMI classes are defined according to the ICD-10 classification as: Underweight (BMI < 19.9 kg/m^2^), Normal (BMI: 20.0–24.9 kg/m^2^), Overweight (BMI: 25.0–29.9 kg/m^2^), Class I Obesity (BMI: 30.0–34.9 kg/m^2^), Class II Obesity = (BMI: 35.0–39.9 kg/m^2^), and Class III Obesity (BMI ≥ 40 kg/m^2^). ^†^ The normal weight class served as the reference group in the logistic regression analysis. Abbreviations: aOR = adjusted odds ratio, OR = odds ratio, CI = confidence interval. Adjusted odds ratios (aORs) with *p*-values < 0.05 were considered statistically significant and are presented in bold.

## Data Availability

The original contributions presented in this study are included in the article. Further inquiries can be directed to the corresponding author.

## Abbreviations

The following abbreviations are used in this manuscript

AFIB	Atrial fibrillation
BMI	Body Mass Index
BP	Blood Pressure
CAD	Coronary Artery Disease
CKD	Chronic Kidney Disease
HF	Heart Failure
NRD	Nationwide Readmission Database
PVD	Peripheral Vascular disease
T2D	Type 2 Diabetes
VFIB	Ventricular Fibrillation

## Appendix A

**Table A1:** STROBE Statement Checklist of items.

	Item No	Recommendation	Page No
Title and abstract	1	(a) Indicate the study’s design with a commonly used term in the title or the abstract	Pg. 1 2
		(b) Provide in the abstract an informative and balanced summary of what was done and what was found	Pg. 1
Introduction			
Background/rationale	2	Explain the scientific background and rationale for the investigation being reported	Pg. 1 2
Objectives	3	State specific objectives, including any prespecified hypotheses	Pg. 2
Methods			
Study design	4	Present key elements of study design early in the paper	Pg. 2
Setting	5	Describe the setting, locations, and relevant dates, including periods of recruitment, exposure, follow-up, and data collection	Pg. 2
Participants	6	(a) Give the eligibility criteria, and the sources and methods of selection of participants	Pg. 2
Variables	7	Clearly define all outcomes, exposures, predictors, potential confounders, and effect modifiers. Give diagnostic criteria, if applicable	Pg. 2 3
Data sources/measurement	8 *	For each variable of interest, give sources of data and details of methods of assessment (measurement). Describe comparability of assessment methods if there is more than one group	Pg. 2 3
Bias	9	Describe any efforts to address potential sources of bias	NA
Study size	10	Explain how the study size was arrived at	NA
Quantitative variables	11	Explain how quantitative variables were handled in the analyses. If applicable, describe which groupings were chosen and why	Pg. 2 3
Statistical methods	12	(a) Describe all statistical methods, including those used to control for confounding	Pg. 2 3
		(b) Describe any methods used to examine subgroups and interactions	NA
		(c) Explain how missing data were addressed	Pg. 2 3
		(d) If applicable, describe analytical methods taking account of sampling strategy	NA
		(e) Describe any sensitivity analyses	Pg. 3
Results			
Participants	13 *	(a) Report numbers of individuals at each stage of study—e.g., numbers potentially eligible, examined for eligibility, confirmed eligible, included in the study, completing follow-up, and analyzed	Pg. 3; Figure 1
		(b) Give reasons for non-participation at each stage	Pg. 3; Figure 1, Table A3
		(c) Consider use of a flow diagram	Pg. 3; Figure 1
Descriptive data	14 *	(a) Give characteristics of study participants (e.g., demographic, clinical, social) and information on exposures and potential confounders	Pg. 4; Table 1
		(b) Indicate number of participants with missing data for each variable of interest	Pg. 3; Figure 1, Table A3
Outcome data	15 *	Report numbers of outcome events or summary measures	Pg. 3 4 5 6; Table 1 and Table 2
Main results	16	(a) Give unadjusted estimates and, if applicable, confounder-adjusted estimates and their precision (e.g., 95% confidence interval). Make clear which confounders were adjusted for and why they were included	Pg. 5 6; Table 2; Table A2, Table A3 and Table A4; Figure 2 and Figure 3
		(b) Report category boundaries when continuous variables were categorized	Table 1 and Table 2; Table A2, Table A3 and Table A4
		(c) If relevant, consider translating estimates of relative risk into absolute risk for a meaningful time period	Pg. 6; Figure 3
Other analyses	17	Report other analyses done—e.g., analyses of subgroups and interactions, and sensitivity analyses	Pg. 6; Figure A1
Discussion			
Key results	18	Summarize key results with reference to study objectives	Pg. 7 8
Limitations	19	Discuss limitations of the study, taking into account sources of potential bias or imprecision. Discuss both direction and magnitude of any potential bias	Pg. 8 9
Interpretation	20	Give a cautious overall interpretation of results considering objectives, limitations, multiplicity of analyses, results from similar studies, and other relevant evidence	Pg. 7 8 9
Generalizability	21	Discuss the generalizability (external validity) of the study results	Pg. 9
Other information			
Funding	22	Give the source of funding and the role of the funders for the present study and, if applicable, for the original study on which the present article is based	Pg. 10

* Give information separately for exposed and unexposed groups. Note: An Explanation and Elaboration article discusses each checklist item and gives methodological background and published examples of transparent reporting. The STROBE checklist is best used in conjunction with this article (freely available on the Web sites of PLoS Medicine at http://www.plosmedicine.org/ (accessed on 30 November 2025), Annals of Internal Medicine at http://www.annals.org/ (accessed on 30 November 2025), and Epidemiology at http://www.epidem.com/ (accessed on 30 November 2025)). Information on the STROBE Initiative is available at www.strobe-statement.org (accessed on 30 November 2025).

**Table A2:** The ICD-10 codes for study variables.

Variables	ICD-10 Codes
Atrial fibrillation	I480, I481, I482, I4891
Acute renal failure	N170, N171, N172, N178, N179
Body Mass Index	Underweight, [BMI] 19.9 or less: Z681 Normal Weight, [BMI] 20.0–24.9: Z6820, Z6821, Z6822, Z6823, Z6824 Overweight, [BMI] 25.0–29.9: Z6825, Z6826, Z6827, Z6828, Z6829 Class I Obesity, [BMI] 30.0–34.9: Z6830, Z6831, Z6832, Z6833, Z6834 Class II Obesity, [BMI] 35.0–39.9: Z6835, Z6836, Z6837, Z6838, Z6839 Class 3 Obesity, [BMI] 40 or greater: Z6840, Z6841, Z6842, Z6843, Z6844, Z6845
Coronary Artery Disease	I2510, I2511, I252, I2582, I2584, Z955, Z951
Chronic Kidney Disease	N183, N184, N185, N186, N189, N19, Z4901, Z4902, Z9115, Z940, Z992, Z4931, Z4932
Cardiogenic shock	R570
Dyslipidemia	E785
Heart Failure	I5020, I5021, I5022, I5023, I5030, I5031, I5032, I5033
Hypertension	I10, I110, I119, I120, I129, I130, I1310, I1311, I132, I150, I151, I152, I158, I159, I674, O10011, O10012, O10013, O10019, O1002, O1003, O10111, O10112, O10113, O10119, O1012, O1013,O10211, O10212, O10213, O10219, O1022, O1023, O10311, O10312, O10313, O10319, O1032, O1033, O10411, O10412, O10413, O10419, O1042, O1043, O10911, O10912, O10913, O10919, O1092, O1093,O111, O112, O113, O119
Peripheral Vascular disease	A5203, I050, I051, I052, I058, I059, I060, I061, I062, I068, I069, I070, I071, I072, I078, I079, I080, I081, I082, I083, I088, I089, I091, I0989, I340, I341, I342, I348, I349, I350, I351, I352, I358, I359, I360, I361, I362, I368, I369, I370, I371, I372, I378, I379, I38, I39, Q230, Q231, Q232, Q233, Z952, Z953, Z954
Smoking	F17200, F17201, F17210, F17211, F17220, F17221, F17290, F17291, Z720, Z87891
Type 2 Diabetes	E08x, E09x, E10x, E11x, E13x, O24.1x, O24.3x, O24.8, O24.9
Ventricular Fibrillation	I4901

**Table A3:** Baseline characteristics of all patients admitted for heart failure with diabetes across different BMI classes.

	BMI Data Available	Missing BMI Data	*p*-Value
	26,199 (28.17%)	66,803 (71.83%)	
Age			
Mean (SD)	66.05 (13.24)	73.32 (12.44)	<0.001
Gender			
Male	12,463 (47.57)	35,526 (53.18)	<0.001
Income *			
Low	9128 (34.84)	22,308 (33.39)	<0.001
Low-middle	7411 (28.29)	18,043 (27.01)	
Middle-High	5919 (22.59)	15,592 (23.34)	
High	3741 (14.28)	10,860 (16.26)	
Comorbidities			
CAD	11,352 (43.33)	37,245 (55.75)	<0.001
Hypertension	19,691 (75.16)	50,831 (76.09)	0.003
Smoking	10,957 (41.82)	25,800 (38.62)	<0.001
Dyslipidemia	12,868 (49.12)	33,157 (49.63)	0.156
PVD	5677 (21.67)	18,657 (27.93)	<0.001
CKD	7317 (27.93)	22,289 (33.37)	<0.001
Hospital Course			
Length of stay (IQR days)	4 (3–7)	4 (2–6)	<0.001

Abbreviations: CAD = coronary artery disease, CKD = chronic kidney disease, PVD = peripheral vascular disease. * Income level was pre-coded in the NRD as “ZIPINC_QRTL”, representing median household income for the patient’s ZIP Code, categorized into four quartiles (Q1 = lowest, Q4 = highest). Cutoffs are updated annually. Additional details are available at: https://hcup-us.ahrq.gov/db/vars/zipinc_qrtl/nrdnote.jsp (accessed on 1 June 2025). Patients with missing age (*n* = 8), income level (*n* = 1467), and mortality (*n* = 22) data were excluded.

**Table A4:** Predictors of in-hospital outcomes among diabetes patients admitted for heart failure patients with diabetes.

		Mortality		Cardiogenic Shock		Ventricular Fibrillation		Atrial Fibrillation		Acute Renal Failure	
		aOR (95% CI)	*p*-Value	aOR (95% CI)	*p*-Value	aOR (95% CI)	*p*-Value	aOR (95% CI)	*p*-Value	aOR (95% CI)	*p*-Value
BMI	Normal	Ref	-	Ref	-	Ref	-	Ref	-	Ref	-
	Underweight	1.81 (1.16–2.81)	0.009	2.08 (1.24–3.50)	0.006	2.38 (0.59–9.61)	0.222	0.78 (0.63–0.96)	0.021	0.90 (0.70–1.15)	0.393
	Overweight	0.99 (0.64–1.54)	0.967	0.97 (0.59–1.60)	0.907	1.60 (0.44–5.88)	0.476	0.90 (0.75–1.08)	0.250	0.99 (0.80–1.23)	0.943
	Class I Obesity	0.56 (0.37–0.85)	0.007	0.62 (0.39–0.99)	0.043	0.39 (0.10–1.51)	0.171	1.08 (0.92–1.27)	0.357	0.92 (0.76–1.11)	0.384
	Class II Obesity	0.38 (0.24–0.59)	<0.001	0.43 (0.27–0.68)	<0.001	0.37 (0.10–1.41)	0.146	1.16 (0.98–1.36)	0.079	0.90 (0.75–1.08)	0.265
	Class III Obesity	0.64 (0.43–0.95)	0.026	0.24 (0.15–0.37)	<0.001	0.41 (0.12–1.40)	0.154	1.39 (1.19–1.62)	<0.001	0.96 (0.80–1.15)	0.640
Age		1.04 (1.03–1.05)	<0.001	0.95 (0.95–0.96)	<0.001	0.96 (0.94–0.98)	<0.001	1.05 (1.05–1.05)	<0.001	1.00 (0.99–1.00)	0.198
Gender	Male	Ref	-	Ref	-	Ref	-	Ref	-	Ref	-
	Female	0.80 (0.66–0.97)	0.021	0.64 (0.52–0.79)	<0.001	0.83 (0.50–1.38)	0.462	0.63 (0.60–0.67)	<0.001	0.89 (0.83–0.95)	<0.001
Income	Low	Ref	-	Ref	-	Ref	-	Ref	-	Ref	-
	Low-Mid	0.98 (0.77–1.24)	0.858	0.96 (0.74–1.24)	0.733	0.63 (0.30–1.30)	0.208	1.13 (1.06–1.21)	<0.001	0.88 (0.82–0.95)	0.001
	High-Mid	1.03 (0.81–1.32)	0.805	1.21 (0.93–1.57)	0.155	1.08 (0.56–2.09)	0.822	1.21 (1.13–1.30)	<0.001	0.97 (0.89–1.05)	0.457
	High	1.00 (0.75–1.32)	0.972	1.26 (0.94–1.69)	0.117	1.75 (0.91–3.38)	0.094	1.35 (1.25–1.47)	<0.001	0.98 (0.89–1.08)	0.709
CAD	No	Ref	-	Ref	-	Ref	-	Ref	-	Ref	-
	Yes	1.23 (1.02–1.49)	0.031	1.69 (1.37–2.08)	<0.001	1.74 (1.03–2.98)	0.040	1.00 (0.95–1.06)	0.909	0.95 (0.89–1.01)	0.110
Hypertension	No	Ref	-	Ref	-	Ref	-	Ref	-	Ref	-
	Yes	0.72 (0.58–0.90)	0.003	0.50 (0.40–0.62)	<0.001	1.18 (0.63–2.19)	0.604	0.90 (0.85–0.96)	0.002	0.96 (0.89–1.04)	0.323
Smoking	No	Ref	-	Ref	-	Ref	-	Ref	-	Ref	-
	Yes	0.82 (0.68–0.99)	0.046	0.76 (0.62–0.92)	0.006	0.60 (0.35–1.02)	0.060	0.92 (0.87–0.97)	0.002	0.99 (0.93–1.05)	0.673
Dyslipidemia	No	Ref	-	Ref	-	Ref	-	Ref	-	Ref	-
	Yes	0.72 (0.60–0.87)	0.001	1.01 (0.82–1.24)	0.949	1.23 (0.73–2.06)	0.439	0.98 (0.93–1.03)	0.414	1.03 (0.97–1.10)	0.311
PVD	No	Ref	-	Ref	-	Ref	-	Ref	-	Ref	-
	Yes	1.07 (0.87–1.32)	0.508	1.98 (1.61–2.43)	<0.001	2.21 (1.32–3.72)	0.003	1.43 (1.34–1.52)	<0.001	1.11 (1.03–1.19)	0.007
CKD	No	Ref	-	Ref	-	Ref	-	Ref	-	Ref	-
	Yes	1.67 (1.37–2.02)	<0.001	1.87 (1.50–2.33)	<0.001	1.02 (0.57–1.80)	0.951	1.06 (0.99–1.12)	0.073	4.88 (4.57–5.21)	<0.001

BMI classes are defined according to the ICD-10 classification as: Underweight (BMI < 19.9 kg/m^2^), Normal (BMI: 20.0–24.9 kg/m^2^), Overweight (BMI: 25.0–29.9 kg/m^2^), Class I Obesity (BMI: 30.0–34.9 kg/m^2^), Class II Obesity = (BMI: 35.0–39.9 kg/m^2^), and Class III Obesity (BMI ≥ 40 kg/m^2^). Abbreviations: aOR = adjusted odds ratio, CAD = coronary artery disease, CI = confidence interval, CKD = chronic kidney disease, PVD = peripheral vascular disease.

**Table A5:** Cox Proportional Hazards models for 1-year mortality and 1-year readmission for HF.

		1 Year Mortality				1 Year Readmission for HF			
		HR (95% CI)	*p*-Value ***	aHR (95% CI)	*p*-Value	HR (95% CI)	*p*-Value ***	aHR (95% CI)	*p*-Value
BMI	Normal	Ref	-	Ref	-	Ref	-	Ref	-
	Underweight	1.41 (0.44–4.60)	0.564	1.58 (0.49–5.15)	0.447	0.92 (0.60–1.41)	0.686	0.93 (0.61–1.43)	0.754
	Overweight	0.64 (0.18–2.28)	0.493	0.75 (0.21–2.68)	0.663	0.89 (0.59–1.33)	0.559	0.85 (0.57–1.28)	0.440
	Class I Obesity	0.41 (0.14–1.19)	0.101	0.59 (0.20–1.73)	0.334	0.75 (0.53–1.06)	0.104	0.74 (0.52–1.05)	0.094
	Class II Obesity	0.48 (0.17–1.36)	0.169	0.73 (0.26–2.07)	0.553	0.70 (0.50–0.99)	0.042	0.71 (0.50–0.99)	0.049
	Class III Obesity	0.46 (0.17–1.25)	0.130	0.80 (0.29–2.18)	0.659	0.65 (0.47–0.91)	0.013	0.68 (0.49–0.96)	0.026
Age		1.03 (1.02–1.04)	<0.001	1.03 (1.02–1.04)	<0.001	1.01 (1.00–1.02)	<0.001	1.00 (1.00–1.01)	0.002
Gender	Male	Ref	-	-	-	Ref	-	-	-
	Female	0.91 (0.71–1.18)	0.490	-	-	1.03 (0.96–1.11)	0.416	-	-
Income	Low	Ref	-	Ref	-	Ref	-	Ref	-
	Low-Mid	1.41 (1.03–1.95)	0.034	1.36 (0.99–1.88)	0.060	1.09 (1.00–1.19)	0.058	1.08 (0.99–1.19)	0.077
	High-Mid	1.25 (0.87–1.80)	0.218	1.17 (0.82–1.69)	0.384	1.16 (1.05–1.27)	0.003	1.15 (1.04–1.26)	0.005
	High	1.48 (1.00–2.19)	0.049	1.36 (0.92–2.02)	0.124	1.06 (0.95–1.19)	0.287	1.04 (0.93–1.16)	0.527
CAD	No	Ref	-	-	-	Ref	-	-	-
	Yes	1.07 (0.82–1.38)	0.626	-	-	1.01 (0.94–1.09)	0.793	-	-
Hypertension	No	Ref	-	-	-	Ref	-	Ref	-
	Yes	0.92 (0.66–1.28)	0.609	-	-	1.36 (1.22–1.51)	<0.001	1.35 (1.21–1.50)	<0.001
Smoking	No	Ref	-	Ref	-	Ref	-	-	-
	Yes	0.79 (0.60–1.03)	0.083	0.89 (0.68–1.17)	0.401	1.00 (0.93–1.08)	0.961	-	-
Dyslipidemia	No	Ref	-	Ref	-	Ref	-	-	-
	Yes	0.56 (0.43–0.73)	<0.001	0.51 (0.39–0.67)	<0.001	1.01 (0.94–1.08)	0.844	-	-
PVD	No	Ref	-	Ref		Ref	-	Ref	-
	Yes	1.26 (0.93–1.71)	0.143	1.16 (0.85–1.59)	0.350	1.11 (1.02–1.21)	0.021	1.06 (0.97–1.16)	0.205
CKD	No	Ref	-	Ref	-	Ref	-	-	-
	Yes	1.82 (1.41–2.35)	<0.001	1.67 (1.29–2.17)	<0.001	0.97 (0.90–1.05)	0.444	-	-

Abbreviations: aHR = adjusted hazard ratio, CAD = coronary artery disease, CI = confidence interval, CKD = chronic kidney disease, HF = heart failure, HR = hazard ratio, PVD = peripheral vascular disease. *** The hazard ratios with a *p*-value cutoff of 0.2 were eligible for inclusion into the adjusted model.
